# Sex and Gender Differences in Alzheimer’s Disease: Genetic, Hormonal, and Inflammation Impacts

**DOI:** 10.3390/ijms25158485

**Published:** 2024-08-03

**Authors:** Zahra Kolahchi, Nicholas Henkel, Mahmoud A. Eladawi, Emma C. Villarreal, Prathik Kandimalla, Anna Lundh, Robert E. McCullumsmith, Elvis Cuevas

**Affiliations:** 1 Department of Neurology, Mitchell Center for Neurodegenerative Diseases, School of Medicine, University of Texas Medical Branch, Galveston, TX 77555, USA; zakolahc@utmb.edu (Z.K.); ecvillar@utmb.edu (E.C.V.); 2 Department of Neurosciences and Neurological Disorders, College of Medicine and Life Sciences, The University of Toledo, Toledo, OH 43614, USA; nicholas.henkel@rockets.utoledo.edu (N.H.); mahmoud.eladawi@utoledo.edu (M.A.E.); prathik.kandimalla@rockets.utoledo.edu (P.K.); anna.lundh@rockets.utoledo.edu (A.L.); robert.mccullumsmith@utoledo.edu (R.E.M.); 3 ProMedica Neurosciences Center, Toledo, OH 43606, USA

**Keywords:** sex differences, Alzheimer’s disease, risk factors, personalized medicine, neuroinflammation

## Abstract

Two-thirds of Americans with Alzheimer’s disease are women, indicating a profound variance between the sexes. Variances exist between the sexes in the age and intensity of the presentation, cognitive deficits, neuroinflammatory factors, structural and functional brain changes, as well as psychosocial and cultural circumstances. Herein, we summarize the existing evidence for sexual dimorphism and present the available evidence for these distinctions. Understanding these complexities is critical to developing personalized interventions for the prevention, care, and treatment of Alzheimer’s disease.

## 1. Introduction

Current estimates indicate that 6.7 million individuals in the United States (US) are afflicted with Alzheimer’s disease (AD) [1,2]; two-thirds of the patients with AD are women [3]. As the population of the US ages, AD and other forms of dementia will become increasingly prevalent. If medical advancements are not produced to prevent, abate, or cure this devastating illness, projection estimates predict that 13.8 million individuals will be diagnosed with AD by 2060 [2]. In 2019, 121,499 deaths in the US were attributed to AD [2]. Among people aged 65 or older in the US, twelve percent of women and nine percent of men have AD [4]. This statistic comprises approximately 7 million people, consisting of 4.1 million women and 2.6 million men [3]. Clearly, sex influences the frequency of AD with greater prevalence in women than men. In this review, we summarize evidence detailing the profound differences in AD between females and males. We describe epidemiological, genetic, neuropathological, hormonal, intestinal (gut microbiota), preclinical, and socio-cultural evidence with an emphasis on the role of neuroinflammation in sex differences in AD.

## 2. Epidemiological Evidence for Sexual Dimorphism in Alzheimer’s Disease

The frequency of AD is greater in women than men in the US [3]. However, there are equivocal reports comparing the incidence and prevalence of AD between females and males when controlled for age [5,6]. While the explanations for these variations are not clear, they suggest that differences in the incidence of AD between the sexes may be geographically and temporally dependent [7].

One European study reported differential incidence rates of AD by sex in those older than 85; in males, incidence decreased, but in females, incidence increased [8]. Two Asian studies reported a similar, albeit non-significant, trend [9,10]. In contrast, the Cognitive Function and Aging Study conducted in the United Kingdom (UK) found that males had a higher incidence rate [11].

Alternatively, several American studies showed that the incidence of AD increased similarly with age in men and women [12,13,14,15]. In addition, a global comprehensive meta-analysis found that the incidence and prevalence of AD was not statistically different between men and women beyond 60 years of age [16].

In epidemiological studies, estimates of prevalence usually derive from cross-sectional surveys conducted in community, institutional, or clinical settings and are vulnerable to survivor and selection biases [17]. For example, the main risk factor for developing AD is age, and, on average, females live longer than males [18,19]. Reporting a higher prevalence of AD in women may result from survival bias rather than sex-specific variables that increase the disease risk [13]. Also, this survival bias should be considered when assessing the incidence rate in men. For instance, males tend to have a higher mortality rate from cardiovascular disease in middle age compared to females [19]. Yet, males who survive beyond 65 have a healthier cardiovascular risk profile, subsequently reducing their risk of developing dementia [19]. Thus, the comparison of the AD risk is between exceptionally robust males and females of average health and longevity [20]. Nonetheless, although survival bias may be a factor, it does not fully explain the sex differences in AD prevalence and incidence [21]. Though it is important to take longevity into account, new research indicates that the higher incidence of AD in women may be due to other components as well [22]. Therefore, biological, social, and behavioral elements all have a role in the variations in brain alterations, AD development, and symptom presentation between the sexes [7,23,24]. The longevity concept ignores certain significant factors. First of all, there is now a difference of less than five years in the average life expectancy in the United States between males and females [25]. Research conducted in Europe indicates that by 2030, the difference in longevity will be less than two years due to the steady increase in male survival rates [26]. Second, statistical models have demonstrated that, even after controlling for gender-dependent mortality rates, age at death, and variations in longevity, women still have a twofold greater incidence and lifetime risk of AD [27,28]. Furthermore, differences in the structure, function, and age-related changes to the brain in men and women are well-documented [27]. According to recent findings, women tend to accrue more tangle load than men do with the same brain Aβ amounts, although there is no difference in the lifetime risk of AD [29]. This finding suggests that the pathogenesis of AD may start sooner in women.

## 3. Genetic Risk Factors Associated with Sexual Dimorphism in Alzheimer’s Disease

Many differences in gene expression have been observed between aging men and women. Genes involved in energy generation are often down-regulated in men, whereas immune response genes are typically up-regulated in women [30]. The apolipoprotein ε 4 (APOEε4) allele is the most powerful genetic risk factor for Late Age Onset Alzheimer’s Disease (LOAD) [31,32], while the APOEε2 allele is protective [32]. In mild cognitive impairment (MCI), the impact of APOEε4 on the risk of AD is sex- and zygosity-specific [33]. In patients aged 65–69 who are homozygous for APOEε4 alleles, females have worse memory in comparison to males, and at ages 70–74, worse global cognition [33]. The APOEε4 allele impairs brain structure and metabolism more in females than in males [34]. With respect to the female AD-related risk genes, bridging integrator 1 (BIN1) has gained recognition as the second most significant susceptibility gene associated with sporadic AD [35,36], and a greater risk of developing AD in females compared to males [37]. BIN1 affects AD pathogenesis through the tau pathway and is overexpressed in the brains of AD patients [38].

### Differential Gene Expression and Pathway Enrichment in Male and Female AD

To demonstrate the differences between male and female subjects with AD, we reanalyzed the reposited Mayo Clinic Alzheimer’s Disease Genetics Studies (MCADGS) dataset. The raw count matrix for RNA-seq of the temporal cortex was acquired from the Synapse web portal, specifically from Project SynID: syn2580853. In total, the analyzed dataset comprised 119 subjects and was split into two subsets based on biological sex information: female (N_CTL_ = 36, N_AD_ = 28) and male (N_CTL_ = 39, N_AD_ = 16). Notably, there was a similar percentage of two allele APOε4 (about 13%) and one allele APOε4 (about 50%) in male versus female subjects in this database. Subsequent analyses were conducted separately for each subset: female AD vs. female CTL and male AD vs. male CTL. Missing patient medical information (PMI) records in the metadata were imputed with a fixed value (−1), followed by conducting a variance partitioning analysis using the variancePartition R package v1.30.2 [39] with the formula ~ Age+RNA integrity (RIN)+postmorteminterval (PMI)+Diagnosis. The analysis aimed to identify and filter out genes that do not explain the variability between the phenotypes. A differential analysis was conducted using the DESeq2 R package v1.40.2 [40], employing the same formula.

**Figure 1:**
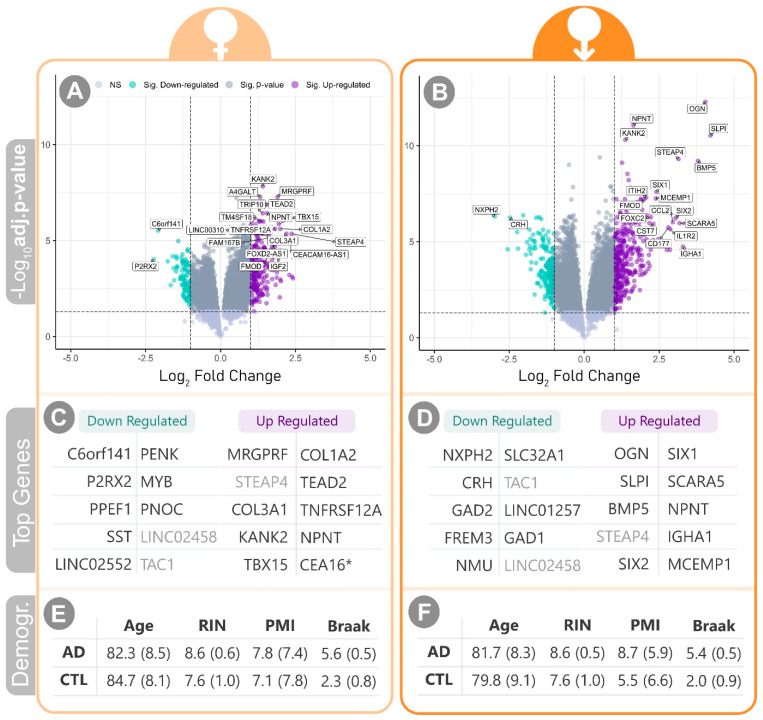
Differential gene expression (DGE) analysis of the sex-based stratified data is presented. The left panel shows the female subjects’ results (female AD vs. female CTL), and the male subjects are represented on the right. (**A**,**B**) show the volcano plots of [–*Log_10_* (*BH adj. p-value*)] vs. [*Log_2_ Fold Change* (*L2FC*)] for the DGE output. The significance thresholds are set to 0.05 for the adj. p-value and ±1 for the L2FC. Green-colored genes are significantly down-regulated, and violet represents significant up-regulation. The top 20 genes—regardless of directionality—based on the value of rank = [–Log_10_ (adj. *p*-value) × L2FC] are labelled. (**C**,**D**) list the top ten down- and up-regulated genes. Lighter-colored symbols are shared between the male and female lists. (**E**,**F**) summarize the demographic data for the cohorts under study. * CEA16: CEACAM16-AS1 & SD: Subjects Demographics.

Figure 1 showcases the top differentially expressed genes in the female and male datasets, along with a summary of the covariates. A pathway enrichment analysis was performed using GSEA (Gene Set Enrichment Analysis) with the fGSEA R package v1.26.0 [41]. A rank value of $\left[-Log_{10}\left(p-value\right)\times L2FC\right]$ was calculated based on the p-value and L2FC (i.e., $Log_{2}\left\{Fold\;Change\left(\frac{CTL}{AD}\right)\right\}$) output from the differential analysis. A curated list of pathways was obtained from the Biological Systems Lab website (https://download.baderlab.org/EM_Genesets/current_release/Human/symbol/ (accessed on 31 July 2024, ID: Human_AllPathways). The analysis of the female dataset yielded 168 significantly (q < 0.05) up-regulated and 36 significantly down-regulated pathways. Among them, 128 up-regulated and 14 down-regulated pathways were uniquely enriched in the female dataset. In the male dataset, 46 up-regulated pathways and 25 down-regulated pathways were significantly enriched, with six and three pathways respectively exclusive to this comparison. Table 1 shows the statistically significant pathways that are distinct between the two comparisons.

Next, we determined the level of association with AD for each pathway in the PubMed database. Utilizing the Bio.Entrez v 1.78 package for Python 3.10 (Python Software Foundation, Washington, DE, USA), the PubMed database was queried for the number of times that any given pathway was mentioned with Alzheimer’s disease, and that numerator was divided by the number of times that the pathway was mentioned in any publication. This result was used as a proxy for how related the pathway was to Alzheimer’s disease. We then quantified the pathway’s relative impact, calculating the Z-score of each pathway against a null distribution of 1000 random pathways that were sourced from the Bader Lab Gene Ontology database with the same Alzheimer’s-relatedness calculation being performed as above. Those Z-scores are represented in the provided table. Higher positive Z-scores indicate a strong association with the AD literature, while Z-scores near zero suggest that the pathway has little prior association with the published literature in the PubMed database.

Notably, most of the top differentially expressed genes, as well as the significantly up-regulated and down-regulated pathways are different in male versus female subjects with AD (Figure 1 and Table 1). This striking contrast suggests markedly different molecular perturbations in male vs. female subjects, which is consistent with prior epidemiological, animal model, and pathophysiological work in AD. Some of the findings highlighted in this exploratory analysis included pathways that were previously associated with AD, including the complement system (https://doi.org/10.1016/j.neuron.2018.10.031), which was up-regulated in male and female subjects, as well as interleukin-10 and interferon signaling (IL-10), which were only up-regulated in female AD patients (cite: https://doi.org/10.1016/S0531-5565(00)00176-5 and https://doi.org/10.3389/fncel.2022.949340) (Table 1). Interestingly, some of the pathways were less associated with AD based on our sematic association with the published literature. Interleukin-4 signaling was only up-regulated in male AD, while syndecan-1 signaling was up-regulated only in female subjects (Table 1). Female, but not male, AD subjects had down-regulation of glutamatergic pathways, suggesting a more severe course with a greater loss of excitatory synapses in female AD patients (Table 1). Taken together, this exploratory bioinformatics assessment of male versus female RNAseq profiles from human AD subjects highlights differences that may only be appreciated when male and female subjects are considered independently.

**Table 1:** RNAseq-derived gender-specific enriched pathways in temporal cortex from female and male AD subjects.

**Up-Regulated Pathways in Female AD**	**Impact Z-Score**	**Up-Regulated Pathways in Male AD**	**Impact Z-Score**
SIGNALING BY TGF-BETA RECEPTOR COMPLEX	0.40	DEVELOPMENT OF URETERIC COLLECTION SYSTEM	−0.06
INTERLEUKIN-10 SIGNALING	2.07	KERATAN SULFATE BIOSYNTHESIS	−0.06
VALIDATED TRANSCRIPTIONAL TARGETS OF AP1 FAMILY MEMBERS FRA1 AND FRA2	−0.07	REGULATION OF COMPLEMENT CASCADE	1.62
SYNDECAN-1-MEDIATED SIGNALING EVENTS	−0.07	IL4-MEDIATED SIGNALING EVENTS	−0.06
PID SYNDECAN 1 PATHWAY	−0.07	AP-1 TRANSCRIPTION FACTOR NETWORK	−0.06
COMPLEMENT SYSTEM IN NEURONAL DEVELOPMENT AND PLASTICITY	6.33		
PID AVB3 INTEGRIN PATHWAY	−0.07		
INTEGRINS IN ANGIOGENESIS	0.25		
INTERFERON ALPHA BETA SIGNALING	2.97		
VALIDATED TARGETS OF C-MYC TRANSCRIPTIONAL REPRESSION	−0.07		
**Down-Regulated Pathways in Female AD**	**Impact Z-Score**	**Down-Regulated Pathways in Male AD**	**Impact Z-Score**
RAS ACTIVATION UPON CA2+ INFLUX THROUGH NMDA RECEPTOR	−0.06	NEUREXINS AND NEUROLIGINS	4.75
NEGATIVE REGULATION OF NMDA RECEPTOR-MEDIATED NEURONAL TRANSMISSION	3.27	SELECTIVE AUTOPHAGY	2.00
AMINE LIGAND-BINDING RECEPTORS	0.71	CILIUM ASSEMBLY	−0.06
SYNAPTIC_VESICLE_TRAFFICKING	6.20		
CRISTAE FORMATION	−0.06		
AEROBIC RESPIRATION I (CYTOCHROME C)	0.47		
PHASE 0—RAPID DEPOLARIZATION	−0.06		
INSULIN RECEPTOR RECYCLING	5.83		
METABOTROPIC GLUTAMATE RECEPTOR GROUP III PATHWAY	1.60		
IONOTROPIC GLUTAMATE RECEPTOR PATHWAY	0.14		

An analysis of the differential gene expression was conducted using the Mayo Clinic Alzheimer’s Disease Genetics Studies (MCADGS) dataset. The raw count matrix for RNA-seq of the temporal cortex was acquired from the Synapse web portal, specifically from Project SynID: syn2580853. N = 119 total subjects: female (N_CTL_ = 36, N_AD_ = 28); male (N_CTL_ = 39, N_AD_ = 16). Subsequent analyses were conducted separately for each subset: female AD vs. female CTL and male AD vs. male CTL. We determined the level of association with AD for each pathway in the PubMed database. Utilizing the Bio.Entrez package for Python, the PubMed database was queried for the number of times that any given pathway was mentioned with Alzheimer’s disease, and that numerator was divided by the number of times the pathway was mentioned in any publication. This result was used as a proxy for how related the pathway was to Alzheimer’s disease. We then quantified the pathway’s relative impact, calculating the Z-score of each pathway against a null distribution of 1000 random pathways that were sourced from the Bader Lab Gene Ontology database with the same Alzheimer’s-relatedness calculation being performed as above. Higher positive Z-scores indicate a strong association with the AD literature, while Z-scores near zero suggest that the specified pathway has less prior association with the published AD literature in the PubMed database.

## 4. Differences in Neuropathology between Human Males and Females with AD

Females and males have different regional frequencies of neurofibrillary tangles (NFTs), particularly when age intervals are included [42]. Female APOEε4 carriers are more likely than male carriers to develop amyloid plaques and NFTs in the early stages of the disease [43]. Additionally, compared to males, females exhibited higher NFT counts in the hippocampus. As such, “hippocampal-sparing-AD” was more frequently found in males, whereas “limbic-dominating-AD” was more frequently found in females [42].

Neuroimaging studies also report that females show greater neuropathology and cognitive decline than males. Females show significantly greater hippocampal atrophy [44,45,46] and a lower brain volume compared to males [7]. Hence, it follows that those females with MCI or AD experience a more rapid decline in memory and functional capacity as well as hippocampal atrophy [45,47]. Females exhibit a twofold decline in overall cognitive function relative to males [48]. This supports a strong association between the extent of the neuropathological burden and cognitive performance in AD, and females are more susceptible than males.

Differences between males and females suggest the involvement of sex hormones. Indeed, the rapid decline in blood levels of ovarian hormones in the mid-life of females can have a profound effect on cognition. It is widely acknowledged that higher levels of estradiol are linked to an improved cognitive performance [49]. In regard to AD, estrogens regulate Aβ and tau [50,51] and decrease neuronal susceptibility to apoptosis when exposed to Aβ, particularly in the hippocampus [52].

## 5. The Role of Sex Hormones in the Development of AD

### 5.1. Ovarian Hormones in the Development of AD

The biochemical and cellular mechanisms of AD start decades prior to the development of clinical signs and symptoms, resulting in a 15- to 20-year prodromal, silent stage that starts during mid-life in both sexes [53]. Coincidentally, menopause in women begins at approximately the same stage of life as the prodromal stage of AD, around the age of 50. Approximately 20 years later is the average age of AD diagnosis, 70 [54,55]. Neuroimaging studies associated the menopausal transition (MT) with the beginning of AD pathology in mid-life, suggesting that the MT is involved in the development of AD [24,56,57]. Elevated Aβ deposition, decreased glucose metabolism, and the loss of white and gray matter volume have been observed in postmenopausal and perimenopausal women, indicating that they have a higher AD burden than premenopausal women and age-matched males [58]. Further evidence for a hormonal role in the increased susceptibility to AD of women comes from a study that reported a 70 percent greater risk of developing AD after ovarian resection and consequent estrogen deprivation [59,60,61]. These results suggest that changes in the circulating levels of ovarian hormones, estrogens in particular play an important role in making women more susceptible than men to AD.

Estrogen receptors have been found in many brain areas, and estrogens regulate a number of physiological mechanisms in the brain, including synaptic plasticity, neuroinflammation, brain macronutrient utilization, blood–brain barrier (BBB) integrity, and docosahexaenoic acid (DHA) metabolism [62,63,64,65]. Possibly, women are more prone to AD because of the profound metabolic changes that take place after menopause. The association between the abrupt decline in estradiol and increased oxidative stress in the brain at the time of menopause may be the consequential factors resulting in initiation of the prodromal phase of AD [66,67]. Female patients with AD, in comparison to age-matched controls, have reduced levels of circulating 17β-estradiol [68].

Women who are peri- or postmenopausal may be prescribed estradiol, progesterone, or a combination, broadly known as hormone replacement therapy (HRT) to boost endogenous ovarian hormone levels to treat unpleasant vasomotor and menopausal symptoms like irritability, depression, perspiration, hot flashes, and urinary incontinence [69]. Epidemiological studies on women’s health conducted in the 1990s revealed that women who received HRT experienced a lower incidence of AD than untreated women [70,71,72]. However, some clinical studies found no beneficial effect of HRT on the development or treatment of AD [73,74,75]. A recent meta-analysis showed negative effects of HRT on cognitive function in women above 60 years of age. Among the included studies, just two investigated women who were under 60 years old: in one of these, no effect of oral estrogens on cognition was reported, and in the other study, conjugated equine estrogen (CEE) and medroxyprogesterone acetate (MPA) had positive benefits on cognition [76]. Another study investigating the modulating effect of age and APOEε4 on the response to HRT [77] found that HRT was associated with better delayed memory and greater entorhinal and amygdala volumes only in APOEε4 carriers [77].

### 5.2. Testicular Hormones as a Risk Factor for Developing AD

Testosterone levels are reduced in male AD patients compared to age-matched healthy males [78,79], and lower levels of testosterone are associated with a greater risk of developing AD and poorer cognitive function in elderly males [80]. Another study showed that free testosterone may decrease the early pathogenic deposition of Aβ in women, as well as slowing the development of AD-specific hippocampal neurodegeneration in men [81]. Testosterone is aromatized into 17β-estradiol in the brain, or into the stronger androgen dihydrotestosterone (DHT) by the enzyme 5-reductase [82]. The actions of testosterone in the brain are amplified to an extent by 17β-estradiol, which activates estrogen receptors (ERs) [83].

Notably, only men are susceptible to prostate cancer, and over 50 percent of prostate cancer patients are now being treated with androgen-deprivation therapy (ADT) following diagnosis [84]. However, one study [85], contrary to others [86], indicates that ADT may increase the risk of developing dementia and cognitive dysfunction.

## 6. Sex-Related Differences in the Microbiota–Gut–Brain Axis

The human gut is home to billions of microbes. A strong correlation between the gut microbiota and the onset and progression of numerous neurological conditions exists, functionally representing the microbiota–gut–brain axis [87,88]. Gut dysbiosis is associated with neurodegenerative diseases such as Alzheimer’s disease, Huntington’s disease, and Parkinson’s disease [89,90,91,92]. Moreover, gut microbiota play a role in regulating cognitive functions like memory and learning [93].

Alterations in the intestinal microbes also contribute to the underlying causes of AD, given that the composition and activity of intestinal microorganisms may impact the pathological processes of dementia- and age-related cognitive decline [94,95,96,97]. Sex disparities in the composition of gut microbiota have been reported, mostly in animal research [98,99]. Some human studies also indicate a strong sex difference in gut microbiota [100,101,102], while others have not observed any significant difference [103,104]. Long term antibiotic administration in mouse models of AD, including APPSWE/PS1ΔE9 and APPPS1-21, has resulted in a decrease in Aβ deposition only in male mice [92,105,106]. Treatment with antibiotics in the early stages of life causes sex-specific microbiome changes that are associated with decreased extracellular Aβ deposition, decreased levels of Aβ peptides that are insoluble in formic acid (FA), changes in the morphology of plaque-associated microglia, and Aβ neurodegenerative features. These effects were particular to male APPPS1-21 mice [107]. Additionally, the fecal microbiota transplantation of APPPS1-21 male mice that were previously treated with antibiotics in their lifespan led to partial restoration of Aβ accumulation, hence confirming causality [92]. Another study [108] examined the sex difference in the impact of the oral administration of probiotics, antibiotics, and synbiotics in the App^NL-G-F^ mouse model of AD. Their results demonstrated different effects of the alterations to gut microbiota between males and females. Among the male and female App^NL-G-F^ models of AD that were given probiotics, only female subjects experienced a decrease in Aβ plaques, microgliosis, and brain TNF-α, as well as improved memory, as compared to control groups that did not receive any treatment [108].

Explaining the contribution of gut microbiota to AD pathology, bacteria-derived amyloids may seep from the intestinal lumen and build up in the brain and systemic circulation [109]. Reactive oxygen species quantities may rise as a result, and nuclear factor-κB (NF-κB) signaling may be activated, up-regulating the proinflammatory microRNA-34a (miRNA-34a). Consequently, miRNA-34a would suppress TREM2 expression (triggering receptors that are expressed in microglial/myeloid cells-2), impairing phagocytosis and causing Aβ 42 peptide buildup [109,110]. The leakiness of the gut can be further aggravated by bacterially produced lipopolysaccharide and amyloids, which can continue to raise the levels of cytokines and proinflammatory molecules that are strongly associated with AD, such as IL-17A and IL-22 [111,112]. These cytokines can penetrate the gastrointestinal tract and then the blood–brain barrier to enter the brain. The cytokines may proceed to initiate immunogenic responses, the release of reactive oxygen species, and the signaling of NF-κB, CD14, and toll-like receptor 2/1, all of which contribute to neurodegeneration [111,113].

## 7. Mood, Cultural, and Social Factors Involved in Sex Differences in AD

Depression is a risk factor for AD dementia in both men and women [114]. Recent estimates suggest that a midlife depression diagnosis may lead to a 70 percent increased risk of developing AD [115]. Females are twice as likely as males to experience depression [116]. Because mood and memory are associated with the same brain regions, depression may impact late-life cognitive function. In the Women’s Health Initiative Memory Study (WHIMS), clinically significant depressive symptoms were associated with a nearly twofold increased risk of MCI and dementia [117]. Given that females have a higher lifelong prevalence of depression, this might be associated with the increased AD frequency [6]. Women exhibited higher mean scores on the NeuroPsychiatric Inventory (NPI) for depression, anxiety, and overall total neuropsychiatric symptoms (NPS) in a study of individuals with newly diagnosed AD dementia who were not receiving treatment for AD or neuropsychiatric symptoms [118]. According to several additional research articles, women who have been diagnosed with AD dementia exhibit more depression symptoms than males. In contrast, agitation is more prevalent in men with AD dementia than in women [119].

It has been reported that a lower level of education is associated with higher risk of dementia in men and women [120,121]. Moreover, engaging in cognitive activities lowers an older person’s chance of dementia [122,123]. Over 10% of the variation in an individual’s cognitive performance may be explained by their intellectual lifestyle, which includes their educational background, occupations, and level of daily cognitive engagement [124].

Thus, females born in the first half of the 20th century may have a higher risk of developing dementia due to their lower educational attainment compared to males [7]. Aside from the direct impacts of education on the risk of dementia-related diseases that some studies have documented, women’s historically lower level of education may also indirectly raise the risk of dementia-related diseases through elevated levels of distress and mental health issues [125]. Furthermore, a cohort study reported that sex is not associated with the incidence rate of AD when controlling for the educational level [126].

Gender norms have shaped career chances historically, with a higher proportion of males than women in the workforce [127]. Furthermore, there has long been a gender gap in occupations, with women being more likely to work in unpaid labor activities like childcare and less likely to hold professional or management positions [128]. Professional or management employment has been associated with a 22% decrease in cognitive decline as well as a 44% reduction in MCI, according to a meta-analysis of nine prospective studies [129].

Males tend to suffer from a more severe course of the disease, leading to early mortality [130,131]. In fact, the male sex is a significant predictor of both an aggressive disease course and progression to death following an AD diagnosis [132]. While male mortality is related to disease factors, such as dementia severity and delirium frequency, female mortality is not. Instead, female mortality is associated with measures of disability, the inability to perform daily tasks, comorbidities, and the presence of pressure sores and malnutrition [130]. One proposed reason for these conditions is that females show resilience to tau pathology, possibly due to their increased immune response compared to males [20].

## 8. Animal Studies Evaluating Sex Differences in AD

### 8.1. Sex Differences in Animal Models of AD

Thus far, we have summarized the evidence in human AD research related to sex differences in the susceptibility to, progression of, and outcome of AD. Sex differences in AD pathology have also been reported in animal models. In summary, female mice exhibit increased neuropathological markers of AD and poorer cognitive outcomes compared to male mice (Table 2).

Several transgenic mouse models are designed to increase Aβ production and deposition [133]. Mutant forms of amyloid precursor protein (APP), presenilin-1 (PS-1), and PS-2 genes are commonly used in these mouse models to induce familial AD [133,134,135]. Studies on APP/PS-1 mice reported higher Aβ levels [133,136,137,138,139], poorer learning and memory [137,140], greater neurodegeneration [136], and a more severe inflammatory microenvironment [136] in female compared to male APP/PS-1 mice. Conversely, Li X. et al. observed more severely impaired glucose and insulin tolerance and higher cholesterol and triglyceride levels in male compared to female APP/PS-1 mice [137]. Meanwhile, Davis et al. studied the effects of X chromosomes on AD pathology in an APP mouse model and found that the second X chromosome reduces neurological deficits and mortality without affecting the levels of Aβ or other protein markers [141]. Furthermore, another AD mouse model, Tg2576, with Swedish mutant human βAPP, expresses excessive amounts of hβAPP and starts to form Aβ plaques at the age of 8- to 10-months [142,143]. Female Tg2576 mice have higher Aβ levels, increased Aβ plaques, and poorer cognitive function than males [144,145].

A triple-transgenic familial mouse model, 3xTgAD, carrying tau_P301L_, PS1M146V, and APPSwe transgenes, was developed to explore the interplay between Aβ and tau and their impact on synaptic function [146,147]. This mouse model generates an AD-like pathology with Aβ and tau accumulations. In human AD patients, Aβ deposition starts in the cortical parts of the brain and subsequently spreads to the hippocampus. Contrastingly, tangle development often starts in the limbic brain region and later spreads to the cortical areas [148]. This is exactly the pattern of development seen in 3xTgAD mice [146]. Compared to male 3xTgAD mice, females showed increased Aβ deposition and greater cognitive deficits in several studies [149,150,151,152,153]. However, one study found comparable cognitive deficits in male and female 3xTgAD mice [154].

It is widely accepted that the APOEε4 allele increases the chance of developing AD [31,155]. Having the potential to be utilized as a possible treatment target for AD, many studies have investigated the connections between sex and APOEε4. Preclinical work suggests that the APOEε4 effects on cognition impairment are modulated by sex and age and are increased in aged female mice [156]. When studying mice that were carrying both genes of familial (3xTg) and sporadic (ApoE4) AD, hippocampal histology showed that female ApoE4/3xTg mice had elevated levels of Aβ proteins, β-site APP cleavage enzyme (BACE1), and Sp1 (BACE1 transcription factor) in comparison to male ApoE4/3xTg, female 3xTg, and nonTg mice [157]. In addition, female ApoE4/3xTg mice showed a more severe AD pathology in the hippocampus and the earlier onset of spatial and memory impairment than male ApoE4/3xTg mice [157].

Studies on EFAD mice, a mouse model of AD homozygousness for knock in ApoE gene and 5xFAD, also showed a sex-dependent effect of the ApoE4 allele [158,159]. Female EFAD mice had a more severe AD pathology in terms of an increased plaque number and decreased plaque compaction due to lowered microglial interactions with Aβ deposits, an elevated level of soluble Aβ, and increased cerebral microbleeds and amyloid angiopathy [158,159]. The ApoE4 allele altered lipid and amino acid metabolism throughout the brain in a sex-dependent manner [160] and likely contributes to AD neuropathology through the impairment of energy, lipid, glucose, and amino acid metabolism in the synaptosomes’ mitochondria [161]. In contrast, another study investigated ApoE4 and E3 male and female mice behaviors and found that only ApoE4/4 females recognized a new object in the novel object recognition (NOR) test [162].

Genetic variations such as P301L and P301S have been found among AD patients with familial tauopathy, which may facilitate tau aggregation to produce paired helical filaments and NFTs [163,164]. In comparison to male P301L-tg mice, female mice showed more body weight loss and a worse survival rate, strongly correlating with the accumulation of tau and p-tau in the regions of the brain which are most impacted by tauopathy [165]. Female mice with mutant APP and P301L had more NFT in all of the brain areas except the pons, compared to their male counterparts [166], yet studies on P301S mutant mouse models indicated a more severe pathology in male mice compared to females [167,168].

Investigating sex differences in a mouse model of FTLD called the TAU58/2 line, which expresses the human 0N4R tau isoform via the P301S mutation, found that male TAU58/2 mice had more NFTs, higher soluble tau levels, and higher quantities of insoluble tau in hippocampal samples than female TAU58/2 mice [167]. Another recent study of the P301S mice model showed that male P301S animals, compared to female transgenic mice, lose weight more quickly and experience more severe dyskinesia and memory impairment. Male P301S mice showed distinct variations in a number of plasma variables, including as MIG, TNF-, IL-13, and IL-10, in comparison to female P301S mice, which was reasonable given the sex disparities in behavior and neuropathology [168].

The 5XFAD mouse model was created in 2006 and expressed excess amounts of human PSEN1 protein with two Familial AD (FAD) mutations (M146L and L286V), as well as human APP with three FAD mutations: the Swedish (K670N, M671L), London (V7171), and Florida (I716V) mutations [169]. The mouse Thy1 promoter contains neural-specific elements that control the expression of both of these transgenes, directing their overproduction only in brain neurons [170]. A recent study focusing on the genotype and sex difference in the 5XFAD mouse model [171] found that females showed greater amounts of human APP and amyloid-β in addition to increased inflammation when compared to their male counterparts. Highlighting that these markers were associated with the hyperactivity that was seen in both sexes, female 5XFAD mice showed minor abnormalities in object and social exploration.

Regardless of their genotype, male mice expressed stress markers and neurotrophic factors more strongly than females, and these traits were associated with an improved cognitive function [171]. Other studies found poorer cognitive performance [172], greater amyloid-β accumulation [173], and a higher plaque burden [173] in female 5XFAD mice. On the other hand, Roddick et al. assessed olfactory-delayed matching-to-sample tasks in 5XFAD mice and observed that female mice performed better, indicating a better working memory [174].

### 8.2. Sex Differences in Neuroinflammation Contributing to AD

Emerging evidence suggests that neuroinflammation has a pivotal role in AD onset and progression [175]. Currently, neuroinflammation is recognized as a main characteristic of AD. There is widespread agreement that inflammation starts at the very beginning of the AD pathology during the prodromal phase, and has both beneficial and detrimental effects [176]. Microglia, as the most prevalent of the phagocytic cells in the brain, can either enhance or inhibit AD progression. Early microglia activation is neuroprotective because the microglia remove soluble Aβ via phagocytosis, micropinocytosis, and Aβ-degrading enzyme-mediated proteolytic degradation [177,178,179]. Nonetheless, the neuroinflammatory pathways in AD involve the activation and expansion of the microglia, which triggers the release of a range of inflammatory mediators [179]. In addition to directly damaging neurons, microglia-mediated neuroinflammation also promotes protein aggregation, which is one of the most notable characteristics of neurodegenerative diseases and plays a role in AD pathogenesis [178]. When Aβ, tau oligomers, and subsequent secretions activate the microglia, they draw in nearby microglia to speed up the active removal of misfolded protein aggregates and degenerated neuronal bodies. As the disease advances, the expression of many components linked to Aβ clearance is down-regulated by the pro-inflammatory cytokines that were generated in response to Aβ aggregation, which encourages even more accumulation of Aβ and neurodegeneration [178].

Most data about sex differences in the microglia come from rodent studies. Early in development, there are variations in the microglial density and shape associated with sex, and although these characteristics vary over the lifespan, some of the differences persist in the adult brain. It has been demonstrated that the amount of microglia in the fetal brains of male and female rats does not change at time points immediately before parturition [180]. But postnatal sex variations in the quantity and shape (and gene expression) of microglia start to show up, and these start to organize the rodent brain shortly after birth. At postnatal day 4 (P4), male rodents have a larger number of microglia than females in the parietal cortex, the amygdala, the CA1, CA3, and dentate gyrus (DG) areas of the hippocampus [180]. Although similar results have been observed in other studies [181], there are studies that report a great number of microglia in the amygdala, hippocampus, and cortex of male rodents [182,183,184]. In addition to the number of microglia, one study showed that there is a significant difference in brain aging in aged female mice compared to age-matched males in terms of microglial activation [185]. This sex difference was associated with an enhanced inflammatory environment in female mice compared to males through the increased expression of inflammatory genes, mostly related to microglia-specific transcripts, especially those involved in the complement system [185]. Moreover, microglia exhibit sex-specific migration rates, [184] and interferon (IFN) γ regulates microglial mobility following an injury (microbleed) in only male rodents and not females [186].

An investigation into the impact of sex and the APOE4 allele on cytokine production by mice’ astrocytes showed that compared to APOE3, mixed-sex APOE4 primary mice’ astrocytes have an elevated basal expression of many pro-inflammatory cytokines, such as IL-6, MCP-1, MIP-1α, TNF-α, IL-1β, and NOS2 [187]. In sex-specific cultures, an APOE4 female primary mouse’s astrocytes had 1.5–2.5-fold higher levels of IL-6, IL-1β, and NOS2 than an APOE4 male’s, and both were higher than an APOE3 primary mouse’s astrocytes [187].

Positron-emission tomography (PET) using the 18 kDa translocator protein (TSPO) has become popular over the past ten years as a method of evaluating microglial activity [188]. Studying female and male patients with AD showed a sex difference in cortical TSPO-PET signals, with a stronger increase in the TSPO-PET signal being observed in prodromal AD females as opposed to prodromal AD males [189]. Although this signal was not associated with Aβ plaques, it could be related to tau accumulations [189]. Male brains’ microglia in post-mortem tissue from AD patients had a uniformly ramified appearance, while the microglia in female brains showed a greatly varied morphology [190]. In addition, men showed a higher microglial density than women [190].

Research conducted in vitro has demonstrated that the executive activities of activated microglia, such as phagocytosis [191], and the release of inflammatory and toxic molecules, including nitric oxide (NO) and the tumor necrosis factor-α (TNF-α), are dependent on Ca^2+^ signaling [192]. Moreover, in vivo models showed that intracellular Ca^2+^ signaling pathways are particularly impaired in microglia that are associated with Amyloid β plaques [193]. Microglia in young brains rarely exhibit Ca^2+^ transients while at rest, yet they consistently react with fast Ca^2+^ signals when a nearby single neuron is damaged [194]. It has been shown that somatic Ca^2+^ transients become more common in microglia as they age and during the amyloid deposition process [193]. Another in vivo study showed that the percentage of active microglia in male mice altered very little through aging, in contrast to female mice, whose percentage of active cells nearly doubled throughout the same period [195]. These functional data indicate the “faster aging” of female microglia and are in line with recent single-cell transcriptome investigations into microglia [196].

**Table 2:** Pre-clinical studies examining sex differences in murine models of Alzheimer’s disease: ↓ means decreased, ↑ means increased, NOR: novel object recognition, BACE1: beta-site amyloid precursor protein cleaving enzyme, MWM: morris water maze, WT: wild-type, PnMS: pre-natal maternal stress, TTR: transthyretin, eIF2α: eukarytoic inititation factor 2 alpha, and NFT: neurofibrillary tangles.

Mice Model	Main Findings (Male vs. Female)
ApoE	ApoE4 Females: perform better NOR task [162], ↓ latency for MWM and vertical exploratory behavior, compared to ApoE3 and WT [156], ↓ metabolic protein expression in synaptosomes and ↑ oxidative stress [161], and ↑ fatty acid/amino acid gene transcription [160].
ApoE4/3xTg	ApoE4 Female: displays earlier onset of spatial and memory impairment [157], ↑ Aβ species, BACE1 and Sp1 [157], and ↑ BACE1 expression [157].
FAD	ApoE4 Female: ↑ microbleeds, cerebral amyloid angiopathy, soluble Aβ [158], ↓ microglial plaque coverage, ↓ TREM2 expression, and ↑ amyloid levels [159].
TASTPM	Females: ↑ Aβ deposition [139].
APP/PS1	Females: ↓ learning and memory [137], earlier onset of conditioned taste aversion deficit [140], ↑ neuronal and synaptic degeneration compared to males [136], w/PnMS ↑ proBDNF but ↓ mBDNF compared to males [197], ↑ Aβ cerebral [133,136,137,138,197], cerebrovascular [136] and peripheral [198], ↑ microhemorrhage, ↑ ptau, ↑ inflammatory cytokines, ↑ astrogliosis, microgliosis [136], ↑ plasma insulin, and ↓ cholesterol [137]. TTR +/− females ↑ Aβ and ↓ testosterone and 17β-estradiol vs. TTR +/+ [199]. Males: ↓ peripheral Aβ [198], earlier glucose and insulin intolerance [128], TTR +/− males equal Aβ, testosterone, and 17β-estradiol vs. TTR +/+ [199]
APP23	Females: ↑ Aβ compared to males but did not respond to Cu^2+^ supplementation though males did [200].
APP	Compared to X0 and XY chromosomal dosage, an additional X chromosome conferred resilience to cognitive decline [141].
Tg2576	Females: ↑ cognitive decline, ↑ soluble Aβ and insoluble Aβ [145], and ↑ senile plaque load and Aβ level [144].
3×Tg-AD	Female: ↑ cognitive deficits [149,150,151,153], ↑ Aβ burden [150,152,153], and ↑ Aβ40 level [154], NFT, and neuroinflammation [153]. Males: ↑ novelty-induced behavioral inhibition [149], demasculinized males: ↑ Aβ burden [150].
5xFAD	Females: higher levels of performance on olfactory delayed matching, compared to males [174], abnormalities in object and social exploration [171], ↑ reversal learning impairment [172], ↑ Aβ, APP, and inflammation [171], under stress condition ↑ neurotoxic Aβ, β-secretase C-terminal fragments, plaque burden, and phospho-eIF2α [173].
P301L	Females: “more severe” synaptopathy and ↑ p-tau [165].
PS19	Males: ↑ monokine expression (IFN-γ, TNF-α, IL-10 and IL-13) and memory impairment compared to females [168].
TAU58/2	Males: ↑ NFTs, soluble, and insoluble tau [167].
TAPP	Female: ↑ NFTs [166].
TgCRND8	Females: learning and memory deficits earlier than males; females did not “overcome” Aβ-associated stereotypies [201].

## 9. Conclusions

While the underlying mechanisms of AD are not fully understood, differences between the sexes, genetic predispositions, hormones, and environmental factors may influence the onset and severity. Thus, it is essential to implement sex-specific approaches in AD research. Historically, the inclusion of females in preclinical studies and sex-specific analyses in clinical studies was limited, and females still experience exclusion from participating in studies in present times, thus causing a delay in the ability to address sex-specific issues. Future studies must consider the sex-specific mechanisms and outcomes of AD in both males and females to approach their treatment and diagnosis more effectively. Such studies may then aid in bridging current knowledge gaps and could lead to improvements in AD diagnosis and treatments that benefit both sexes.
